# Severe Pyothorax Caused by Streptococcus pseudoporcinus and Prevotella buccae: A Case Report

**DOI:** 10.7759/cureus.85770

**Published:** 2025-06-11

**Authors:** Yuki Chiko

**Affiliations:** 1 Department of Internal Medicine, Fukuyama Minami Hospital, Fukuyama, JPN; 2 Department of General Medicine, Okinawa Prefectural Yaeyama Hospital, Ishigaki, JPN

**Keywords:** pleural fluid culture, prevotella buccae, pyothorax, streptococcus pseudoporcinus, β-hemolytic streptococci

## Abstract

Pyothorax is a condition characterized by the accumulation of purulent pleural effusion and is often secondary to pneumonia or lung abscess. The identification of causative pathogens is essential for treatment; however, the sensitivity of pleural fluid culture remains low. We report a rare case of severe pyothorax requiring mechanical ventilation in which *Streptococcus pseudoporcinus *and *Prevotella buccae *were isolated from pleural fluid. This case highlights the potential for β-hemolytic streptococci to cause rapidly progressive and severe infections, even when rarely reported as causative organisms of pyothorax.

## Introduction

Pyothorax is defined as the presence of visible pus in the pleural cavity and is typically a complication of pneumonia or lung abscess, accounting for approximately 80% of cases [1]. The identification of the causative microorganism is crucial for treatment planning. However, the sensitivity of standard pleural fluid culture is reported to be only 14%-21%, and many cases are treated empirically without definitive pathogen identification [2,3]. We report a rare case of pyothorax with severe respiratory failure, in which *Streptococcus pseudoporcinus* and *Prevotella​​​​​​​ buccae* were isolated from pleural fluid culture.

## Case presentation

A man in his 60s presented with a one-month history of persistent cough and left-sided chest pain. Despite outpatient treatment at a local clinic, his symptoms progressively worsened, prompting referral to our hospital. The patient's medical history included chronic pancreatitis and diabetes mellitus; however, he had not been receiving regular treatment, as he had voluntarily discontinued hospital visits. Consequently, the timing of the onset of these conditions remained unclear.

On presentation, the patient's vital signs were as follows: body temperature, 35.5°C; heart rate, 134 beats per minute; blood pressure, 93/67 mmHg; respiratory rate, 58 breaths per minute; and oxygen saturation, 81% on room air. He was alert. Physical examination revealed decreased breath sounds in the left lung field, with no jugular venous distension or peripheral edema. Laboratory findings are summarized in Table 1.

**Table 1:** Laboratory findings. WBC, white blood cell; Hb, hemoglobin; Hct, hematocrit; PLT, platelet; APTT, activated partial thromboplastin time; PT, prothrombin time; INR, international normalized ratio; Glu, glucose; T-Bil, total bilirubin; AST, aspartate aminotransferase; ALT, alanine aminotransferase; LDH, lactate dehydrogenase; GTP, guanosine triphosphate; BUN, blood urea nitrogen; Cr, creatinine; CRP, C-reactive protein; BNP, b-type natriuretic peptide

	Values	Unit	Reference range
Hematology			
WBC	25410	/μL	3300-8600
Hb	13.4	g/dL	13.7-16.8
Hct	30.6	%	40.7-50.1
PLT	56.0	×10^4^/μL	15.8-34.8
Biochemistry			
Glu	214	mg/dL	73-109
T-Bil	0.4	mg/dL	0.4-1.5
AST	23	IU/L	13-30
ALT	21	IU/L	10-42
LDH	336	IU/L	124-222
γ-GTP	138	IU/L	13-64
BUN	26.5	mg/dL	8-20
Cr	1.65	mg/dL	0.65-1.07
Na	137	mEq/L	138-145
K	4.5	mEq/L	3.6-4.8
Cl	95	mEq/L	101-108
Ca	9.5	mEq/L	8.8-10.1
Serology			
CRP	41.94	mg/dL	0.00-0.14
BNP	94.4	pg/mL	0.00-18.4
Blood coagulation test			
APTT	47.3	Seconds	24.0-36.0
PT	15.8	Seconds	10.0-12.0
PT-INR	1.25		0.90-1.10
D-dimer	2.8	μg/dL	0.0-1.0

**Figure 1:**
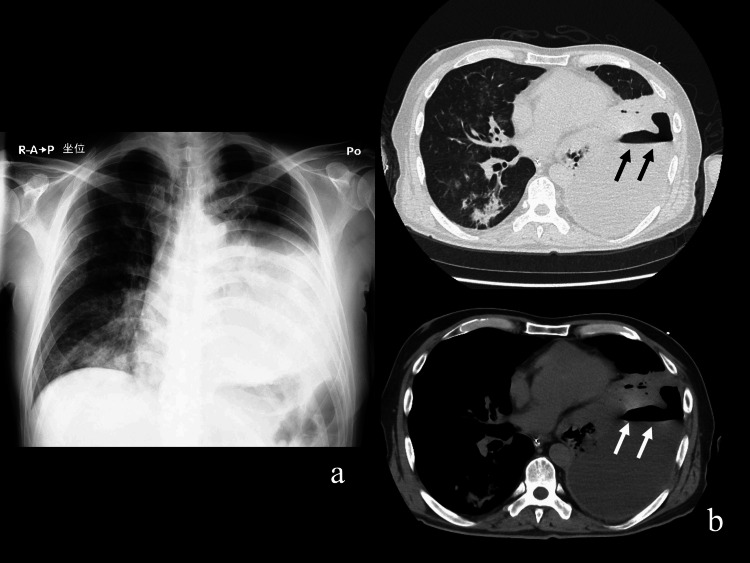
Imaging studies on admission. (a) Chest X-ray showing decreased radiolucency in the left thoracic cavity, suggestive of pleural effusion. (b) Chest computed tomography (CT) revealing a large left-sided pleural effusion with gas formation (arrow), consistent with empyema.

**Figure 2:**
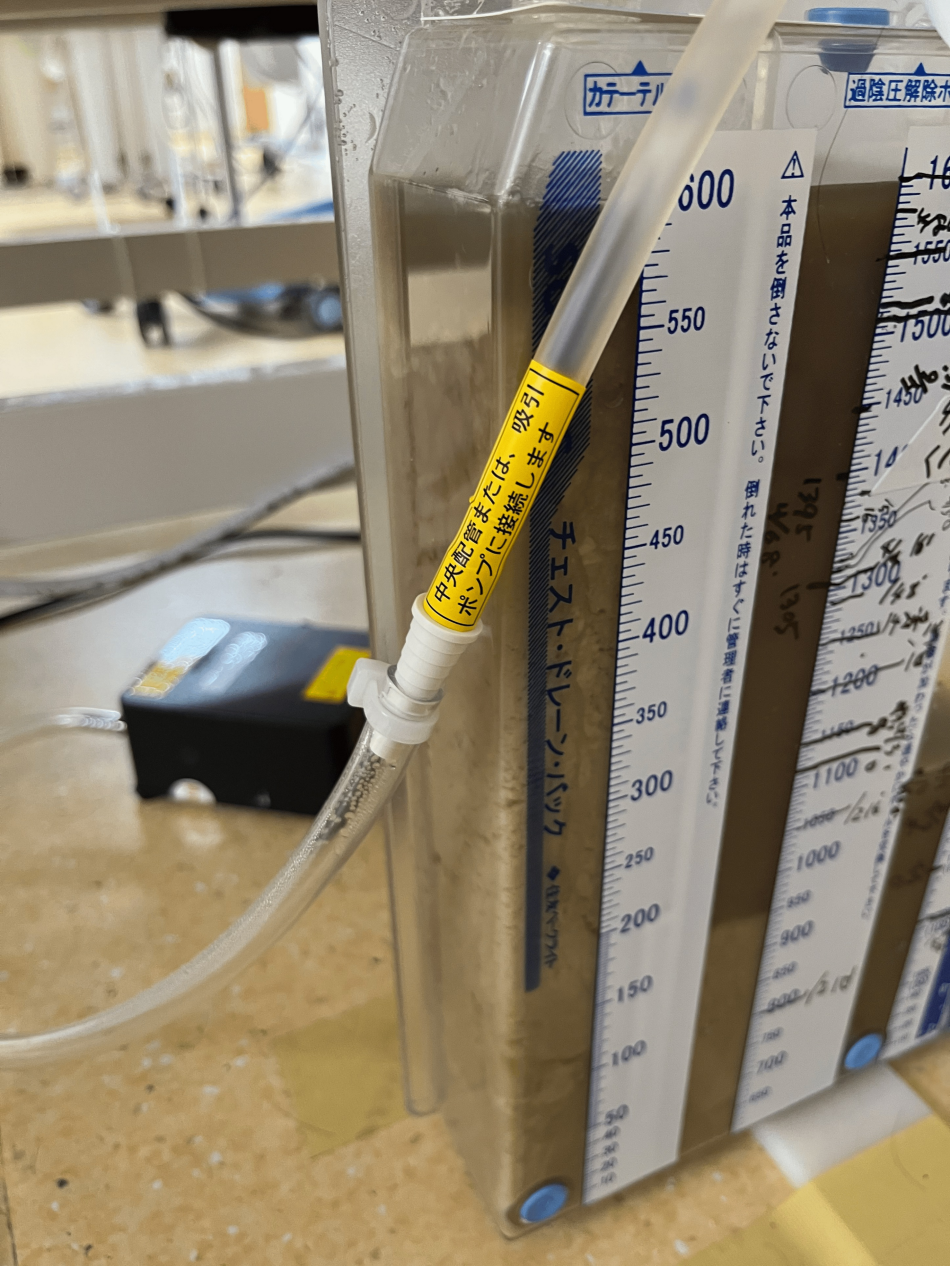
Pleural drainage bag. A large amount of purulent drainage with a foul odor has accumulated.

**Figure 3:**
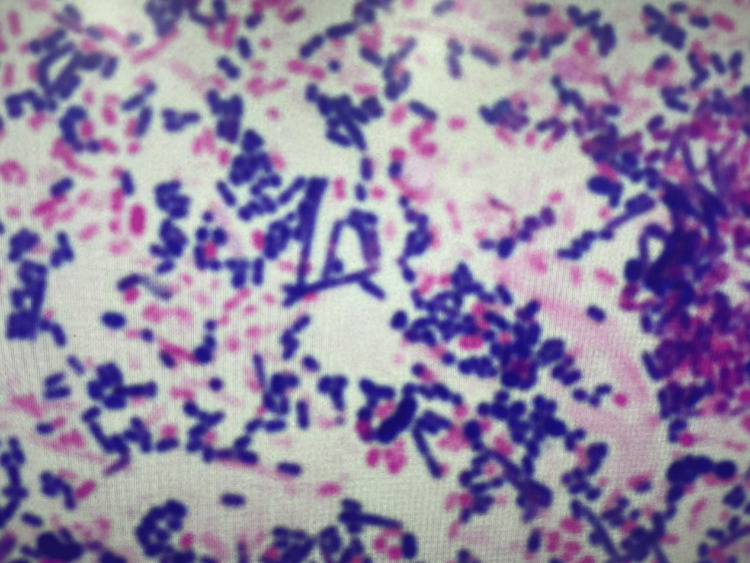
Gram staining of pleural effusion. Multiple bacterial species are observed.

Chest X-ray and computed tomography (CT) revealed a massive left-sided pleural effusion with pleural thickening and suspected gas formation (Figure 1). Due to severe respiratory failure, mechanical ventilation was initiated. Upon admission to the intensive care unit (ICU), chest tube drainage was performed, yielding a large amount of malodorous, turbid, purulent fluid (Figure 2). The pleural fluid analysis is summarized in Table 2. Gram staining demonstrated polymicrobial organisms (Figure 3). As the identification of the causative organism was considered important, the pleural fluid sample was inoculated into a blood culture bottle in addition to being submitted for a pleural fluid culture test. A routine blood culture and sputum culture were also obtained.

**Table 2:** Findings of pleural effusion. %Neu represents the proportion of neutrophils among the total nucleated cells in the pleural fluid. The turbid appearance, decreased pH, low glucose levels, and other findings were suggestive of pyothorax. LDH, lactate dehydrogenase; Glu, glucose; %Neu, percent neutrophils

Appearance	Purulent	
pH	6.86	
LDH	554	IU/L
Glu	1	mg/dL
%Neu	100	%

**Figure 4:**
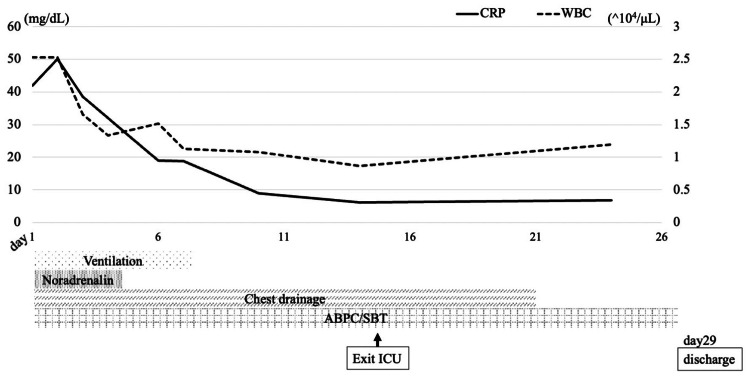
Clinical course. ABPC/SBT, ampicillin/sulbactam; ICU, intensive care unit; CRP, C-reactive protein; WBC, white blood cell

Empiric antibiotic therapy with ampicillin/sulbactam (3 g every six hours) was initiated for the treatment of pyothorax. A few days after admission, *S. pseudoporcinus* and *P. buccae* were identified as the causative pathogens from two sets of blood culture bottles with pleural fluid cultures. The standard blood cultures remained negative. Sputum culture revealed multiple bacterial species suggestive of aspiration; however, no single predominant organism was identified. The susceptibility results for each of the bacteria are shown in Table 3. Gram staining revealed a large number of bacteria, and ampicillin/sulbactam was continued without de-escalation, considering the possibility of a mixed bacterial infection involving organisms other than these two species. The patient's condition gradually improved with continued antibiotic therapy. Circulatory support with noradrenaline was discontinued on hospital day 4. The patient was successfully extubated on day 8, the chest drain was removed on day 21, and he was discharged on day 29 after transitioning to oral antibiotic therapy using amoxicillin/clavulanic acid (Figure 4).

**Table 3:** Susceptibility results for each of the bacteria. PCG, penicillin; AMPC/CVA, amoxicillin/clavulanic acid; ABPC/SBT, ampicillin/sulbactam; CTX, cefotaxime; CFPM, cefepime; AZM, azithromycin; LVFX, levofloxacin; CTRX, ceftriaxone; MINO, minocycline; IPM/CS, imipenem/cilastatin; MEPM, meropenem; VCM, vancomycin; EM, erythromycin; CLDM, clindamycin; ST, sulfamethoxazole-trimethoprim; PIPC, piperacillin; PIPC/TAZ, piperacillin-tazobactam; CMZ, cefmetazole; MFLX, moxifloxacin; MIC, minimum inhibitory concentration; S, sensitive; R, resistant

*Streptococcus* *pseudoporcinus*			*Prevotella* *buccae*		
	MIC			MIC	
PCG	≤0.03	S	PCG	≤0.06	S
ABPC	≤0.12	S	ABPC	≤0.25	S
AMPC/CVA	≤1		PIPC	≤4	S
ABPC/SBT	≤0.12		ABPC/SBT	≤1	S
CTX	0.25	S	PIPC/TAZ	≤4	S
CFPM	0.5	S	CMZ	1	
AZM	≤0.12	S	CTX	≤2	S
LVFX	0.5	S	VCM	>16	
CTRX	≤0.25	S	IPM/CS	≤0.5	S
MINO	≤0.12		MEPM	≤0.5	S
IPM/CS	≤0.12		CLDM	＞8	R
MEPM	≤0.25	S	MFLX	1	S
VCM	0.5	S			
EM	≤0.12	S			
CLDM	≤0.25	S			
ST	≤10				

## Discussion

Standard pleural fluid cultures have low sensitivity, often resulting in empirical treatment without identifying the pathogen [2,3]. The use of blood culture bottles for pleural fluid has been reported to increase the diagnostic yield up to 24%-58.5%, raising positive rates by approximately 15% [4]. In this case, the causative organism was identified through conventional culture; however, efforts to identify the pathogen should not be neglected, especially in severe cases. Pleural fluid culture using a blood culture bottle may be a useful option in such situations. In this case, two microorganisms were identified as the causative agents of the pyothorax. In cases of community-acquired empyema where the causative organisms are identified, the *Streptococcus milleri *group species account for approximately one-third of the cases, followed by anaerobic bacteria and *Streptococcus pneumoniae* [5]. The two bacterial species identified in this case are rare causes of empyema, and in particular, there are very few reports of *S. pseudoporcinus* as a causative pathogen.

The genus *Prevotella* comprises obligate anaerobic, Gram-negative, short rod-shaped bacteria that are part of the endogenous flora of the oral cavity and urogenital tract [6]. On the other hand, *S. pseudoporcinus *is a relatively recently identified β-hemolytic streptococcus, known to colonize the female genital tract [7]. The infection route in our case remained unclear due to the absence of sexual exposure, but pleural fluid culture confirmed its role as the pathogen. Reports of *S. pseudoporcinus* remain extremely limited to date, with documented human infections including skin and soft tissue infections [8], pregnancy-related bacteremia [9], infective endocarditis [10,11], and spontaneous bacterial peritonitis [12]. Only one previous case of empyema was reported, and that case also presented with severe disease [13]. Group A β-hemolytic streptococci are known to cause a rapidly progressive form of pleuritis/empyema termed "explosive pleuritis" [14]. Given that *S. pseudoporcinus* is also a β-hemolytic streptococcus, it may explain the severe disease course in this patient.

## Conclusions

*Streptococcus* *pseudoporcinus* is an uncommon pathogen, and its role in pyothorax remains poorly understood due to the scarcity of reported cases. This case illustrates that *S. pseudoporcinus*, in conjunction with anaerobic organisms such as *P. buccae*, can lead to fulminant pyothorax and septic shock. Clinicians should be aware of this potential pathogen, especially in polymicrobial infections with a severe clinical course. Accurate microbial identification and the prompt initiation of appropriate antibiotic therapy are essential for patient recovery. Further accumulation of such cases is needed to better elucidate the pathogenicity and clinical characteristics of *S. pseudoporcinus*.
